# Coupling machine learning and epidemiological modelling to characterise optimal fungicide doses when fungicide resistance is partial or quantitative

**DOI:** 10.1098/rsif.2022.0685

**Published:** 2023-04-19

**Authors:** Nick P. Taylor, Nik J. Cunniffe

**Affiliations:** Department of Plant Sciences, University of Cambridge, Cambridge, UK

**Keywords:** fungicide resistance, epidemiology, machine learning, qualitative resistance, quantitative resistance, partial resistance

## Abstract

Increasing fungicide dose tends to lead to better short-term control of plant diseases. However, high doses select more rapidly for fungicide resistant strains, reducing long-term disease control. When resistance is qualitative and complete—i.e. resistant strains are unaffected by the chemical and resistance requires only a single genetic change—using the lowest possible dose ensuring sufficient control is well known as the optimal resistance management strategy. However, partial resistance (where resistant strains are still partially suppressed by the fungicide) and quantitative resistance (where a range of resistant strains are present) remain ill-understood. Here, we use a model of quantitative fungicide resistance (parametrized for the economically important fungal pathogen *Zymoseptoria tritici*) which handles qualitative partial resistance as a special case. Although low doses are optimal for resistance management, we show that for some model parametrizations the resistance management benefit does not outweigh the improvement in control from increasing doses. This holds for both qualitative partial resistance and quantitative resistance. Via a machine learning approach (a gradient-boosted trees model combined with Shapley values to facilitate interpretability), we interpret the effect of parameters controlling pathogen mutation and characterising the fungicide, in addition to the time scale of interest.

## 1. Introduction

Plant pathogens have a significant impact on global food production [1,2]. Diseases caused by plant pathogens routinely lead to large losses in crop yields [3]; an estimated 20% of losses of global crop production are caused by disease [4]. However, more than 900 million people are undernourished [5], and food production will need to increase by an estimated 60% by 2050 [1]. Fungal plant diseases can be particularly damaging due to the potential for prolific spore production, long-range spore dispersal, high mutation rates and potential for both sexual [6] and asexual reproduction [7]. Further, fungal pathogens are strongly influenced by climatic factors, so changes in climate are likely to affect the incidence and severity of many diseases caused by fungi [8]. Modern agricultural ecosystems offer reduced species diversity when compared to natural ecosystems, but higher host and pathogen density, creating highly conducive environments for rapid dispersal and evolution of fungal plant pathogens [9]. Current control mechanisms rely strongly on chemical control from fungicides. However, their control is regularly challenged by fungicide resistance due to the enormous evolutionary potential of fungal pathogens.

Control of fungal pathogens is extremely economically important; approximately 16 billion US dollars are spent every year on fungicides globally [4]. Mathematical modelling can be an invaluable tool enabling us to understand the complicated mechanisms underlying fungicide resistance development [10,11]. Theoretical and modelling studies offer numerous advantages to complement experimental studies. For example, field trials are frequently very expensive and it can be difficult to control for confounding factors such as environmental variability between years. The time scales involved in fungicide resistance studies can be extremely long, which leads to increased cost and enormous delays in obtaining results when compared with theoretical studies. Further, fungicide resistance is often (at least initially) present at frequencies so low where it can be difficult to obtain accurate measurements.

Most fungicide resistance modelling studies address qualitative resistance (i.e. where a single mutation causes fungicide resistance) [12–19]. However, many contemporary fungicides in use (e.g. the azole and succinate dehydrogenase inhibitor fungicides [20,21]) are challenged by quantitative resistance (i.e. where several successive mutations are needed to acquire considerable levels of resistance, meaning many strains can be present each with their own level of resistance [17,22,23]). Many questions in the fungicide modelling literature about resistance management have only been addressed in the case of qualitative resistance.

One of the key questions about fungicide resistance management concerns fungicide dose; when should low doses be used in favour of high doses? The vast majority of experimental and modelling literature suggests that high doses select more strongly for qualitative resistance [24], due to increased selection pressure in line with the so-called governing principles of fungicide resistance [25]. This means that low doses are often preferable when tackling qualitative resistance, so long as adequate yield can be maintained.

In theory, high doses may be useful in targeting diploid pathogens [24]. To illustrate this, consider a population containing homozygote sensitive (denoted SS), homozygote resistant (denoted RR) and heterozygote individuals (denoted RS). If resistance is rare, and mating random, then it is most likely that the SR and RR strains will mate with a homozygote sensitive (SS) individual, resulting in more homozygote sensitive or heterozygote individuals. If heterozygote individuals are more susceptible than homozygote resistant individuals then the heterozygote individuals can be better controlled by high doses. This allows higher doses to remove resistant alleles from the population [24]. However, most fungal pathogens are haploid or largely clonal meaning that low doses are usually recommended [24,25].

Partial resistance occurs when the resistant strain is still partially suppressed by the fungicide (as opposed to complete resistance, where the resistant strain is completely unaffected by the fungicide). If dose–response curves converge at high doses, which might occur with partial resistance, then high doses could result in reduced selection for resistance [24–26]. Dose–response convergence means that the difference between infection rates of resistant and sensitive strains is smaller for high doses than at smaller or intermediate doses. Whether this happens depends on the shape of the dose–response curve. This can lead to reduced selection for fungicide resistance at high doses compared to lower doses.

Two types of partial qualitative resistance are described in [17]: Type 1 where resistance is characterised in terms of the maximum fungicide effect; Type 2 where resistance is characterised in terms of the ‘slope’ of the fungicide effect with dose. For Type 1 partial resistance, high doses were found to accelerate resistance emergence, whereas for partial resistance Type 2 there was a minimum in emergence time for intermediate doses, suggesting low or high doses should be favoured. The driver of the result is whether the dose–response curves converge at high doses, which happens with partial resistance Type 2 but not with Type 1.

Models often address the so-called ‘emergence phase’ or the ‘selection phase’ of fungicide resistance. The emergence phase is when resistance arises in a previously sensitive population through mutations [27]. The selection phase is when resistant genotypes are present in a population and subsequently selected for by fungicide use [27]. Most modelling studies focus solely on either the emergence phase [17,28] or the selection phase [13–15].

Although [17] address partial resistance, the model targets only qualitative resistance, and so considers two pathogen strains only. This means that the model does not adequately address the wide range of pathogen strains possible in the quantitative resistance case. However, dose–response convergence is possible when resistance is quantitative as well as when it is qualitative but partial, meaning that high doses may result in reduced selection at higher doses. While [29] defined a theoretical model of polygenically controlled (i.e. quantitative) fungicide resistance, it was not fitted to field data and did not have a notion of crop yield. The model in [30] addresses quantitative resistance parametrized for control of Septoria tritici blotch (caused by *Zymoseptoria tritici*), the most prevalent disease of wheat worldwide [31], using the azole fungicide ‘prothioconazole’. In [30], applications were always at full dose; in this work, we extend the model to consider lower doses and explore the effect of dose choice on disease control and resistance management. We also consider a range of epidemiological and fungicide parameter choices, to allow us to explore beyond the wheat-septoria/azole system.

Many models of fungicide resistance development neglect to explicitly model pathogen mutation [12–17], despite its important evolutionary role [32]. The model in [30] includes pathogen mutation and is capable of modelling both emergence of resistance and selection for resistant strains present in the population. We will explore the effect of parameters controlling the pathogen mutation rate and mutation scale on the optimal dose recommendation.

Some previous plant disease modelling work makes use of sensitivity analyses to explore the effects of different model parameters on the model output. In [33], polynomial regression was used to characterise the epidemiological model output in terms of six model parameters. Sobol’s method was used to evaluate which features have the strongest impact on the model. In this work, we introduce an alternative method, common in machine learning, to explain the effect of eight model parameters on the output of the quantitative resistance model. Machine learning approaches are becoming increasingly important in a wide variety of applications ranging from fraud detection to speech recognition. Gradient-boosted trees models are one such algorithm capable of excellent results on a wide range of problems, often outperforming polynomial regression models, random forests and neural networks [34]. We combine a gradient-boosted trees model with Shapley values [35,36], a technique from game theory for allocating importance to members of a coalition (in our case, variables in a model). This allows us to rank and understand the impact of different parameters on the output of our model, i.e. the optimal dose. We use this approach to explore more clearly the relationships between each model parameter and the optimal dose. The approach facilitates model interpretability and explanation while making use of the accuracy of gradient tree boosting.

In this paper, we address the following questions:

— Can high fungicide doses ever outperform low doses, in terms of yield, when repeatedly applied over many years?
— How does this depend on whether fungicide resistance is qualitative or quantitative?
— What is the effect of fungicide efficacy and pathogen mutation on optimal dose choice over different time scales?
— Do Shapley values help make a complex parameter scan (of the type common in plant disease epidemiology) more interpretable?
— When, and why, do low doses outperform high doses in terms of yield, or high doses outperform low doses?

## 2. Methods

### 2.1. Model explanation

We use the model of quantitative fungicide resistance from [30]. It addresses a diverse pathogen population containing strains which have different sensitivities to a fungicide. These are described by a continuous ‘trait value’ *k* taking values in [0, 1], where *k* = 0 corresponds to a fully sensitive pathogen strain (completely suppressed by a fungicide application) and *k* = 1 corresponds to a fully resistant pathogen strain (completely unaffected by a fungicide application).

The model tracks healthy/susceptible leaf tissue (denoted by *S*(*t*), where *t* is the time within a single growing season) and leaf tissue infected by strain *k* (denoted by *I*(*k*, *t*)). The model is applied over multiple growing seasons. It includes mutation, a host growth function *g*(*t*) and a senescence function Γ(t). The infection rate *β*(*k*, *t*) depends on the pathogen strain *k*, since each strain responds differently to a fungicide application. We refer the reader to [30] for a full explanation of the model derivation and details on the model fitting process. Although the full form of the fitted model as presented in [30] included the effect of disease-resistant crop varieties, for simplicity here we do not model host plant protection, meaning we use the so-called ‘fungicide only’ form of the model from [30].

The model is defined as

2.1 $$\frac{dS(t)}{dt}=g(t)-\Gamma (t)S(t)-S(t)\int_{0}^{1}\int_{0}^{1}\beta (j,t)I(j,t)P(k,j)\;dj\;dk$$

and

2.2 $$\frac{dI(k,t)}{dt}=S(t)\int_{0}^{1}\beta (j,t)I(j,t)P(k,j)dj\;\text{f}or\;k\in [0,1].$$

Variable and function definitions are given in table 1, and the functions *g*(*t*), Γ(t), *P*(*k*, *j*) and *β*(*k*, *t*) are described in more detail below. Parameter values as fitted in [30] are found in electronic supplementary material, appendix 1 table S1.

**Table 1.** List of variables/functions used in the model. Default values are given in electronic supplementary material, table S1. Where it is non-obvious, variable meanings are given.

variable	description	meaning	equation
*k*	fungicide sensitivity trait	describes the different pathogen strains	—
*t*	time (within–season)	—	—
*S*(*t*)	susceptible host tissue	—	2.1
*I*(*k*, *t*)	infected host tissue	—	2.2
*A*(*t*)	total host tissue	—	—
*β*_0_	pathogen infection rate (no fungicide)	baseline infection rate	2.3
*β*(*k*, *t*)	pathogen infection rate (fungicide applied)	depends on time (*t*) and which pathogen strain (*k*)	2.3
*ω*	fungicide asymptote	the reduction in pathogen growth rate at a hypothetical infinite fungicide dose	2.3
*θ*(*k*)	fungicide curvature	how the dose–response curve changes with concentration. different *θ* value for each strain *k*	2.3
*C*(*t*)	fungicide concentration	—	2.4
Λ	fungicide decay rate	how quickly the fungicide concentration decays (exponentially)	2.4
*μ*	pathogen gamma initial distribution mean	controls the distribution of curvature values	electronic supplementary material, appendix 3 equation 1
*b*	pathogen gamma initial distribution rate	controls the distribution of curvature values	electronic supplementary material, appendix 3 equation 1
*g*(*t*)	host growth function	describes wheat growth within the season	2.5
*r*	host growth rate	parameter in host growth function	2.5
Γ(t)	host senescence function	describes senescence of wheat within the season	2.6
*P*(*k*_1_, *k*_2_)	probability of mutation from parent *k*_1_ to offspring *k*_2_	—	2.7
*p*_*M*_	mutation proportion	proportion of offspring that mutate	2.7
*σ*^2^	mutation scale	relates to the magnitude of the change in phenotype when mutation occurs	2.7

#### 2.1.1. Infection rate/effect of fungicide

Denote the fungicide concentration at time *t* by *C*(*t*). Then the infection rate, *β*(*k*, *t*), of strain *k* at time *t* as defined in [30] is

2.3 $$\beta (k,t)=\beta_{0}[1-\omega +\omega \exp(-\theta (k)C(t))],$$

where *θ*(*k*) = −log (*k*) is the so-called fungicide ‘curvature’ parameter for strain *k*, and *ω* is the fungicide asymptote parameter. [30] only considered the case where *ω* = 1, which corresponds to zero growth rate at a theoretical infinite dose (NB the maximum dose is 1 not infinity). When the concentration is 0, the exponential term is 1, so when there is no fungicide present all strains behave identically, i.e. there is no fitness cost to resistance. When the fungicide is applied the infection rates of strains with lower *k* values are suppressed more than that of strains with higher *k* values. This equation is illustrated in electronic supplementary material, appendix 1 figure S1.

The fungicide concentration *C*(*t*) is assumed to decay exponentially after each application with time *t* at rate Λ (electronic supplementary material, appendix 1 table S1). The fungicide concentration equation is

2.4 $$\frac{dC(t)}{dt}=-\Lambda C,$$

where *C* is initially 0 but increases instantaneously by an amount *D* every time a dose of *D* of the fungicide is applied. In [30], only a full dose of 1 was considered. In this work, we consider lower doses, using 10 dose choices: 0.1,0.2,…1. In this work, we restrict our attention to fungicide application programmes containing two sprays at the spray times conventionally called *T*_2_ and *T*_3_ (table 2) [19]. This equation is illustrated in electronic supplementary material, appendix 1 figure S1.

**Table 2.** Model running times, as found in [30]. Note that ‘dd’ refers to the units ‘degree-days’. Degree-days are calculated based on a base temperature of 0°C, and the growing season average temperature in Cambridge (UK) during 1984 to 2003 of 15.2°C (i.e. one calendar day equals 15.2 degree-days), as in [19]. Note that the start time is at growth stage 32, and the host growth and epidemic progress until this point is scaled into the model initial conditions as in [30].

parameter	growth stage	time (dd)	purpose
*t*_start_ = *T*_1_	32	1456	start time
*T*_2_	39	1700	application time 1
*T*_3_	61	2066	application time 2
*t*_end_	75	2515	end time
*T*_*GS*61_	61	2066	start of senescence in [12]
*T*_*GS*87_	87	2900	end time in [12]

#### 2.1.2. Host growth and senescence

Within the season, the host is assumed to initially grow before a period of senescence. We use the same forms for growth, and for senescence, as in [12,15,18]. Let *A*(*t*) be the total amount of tissue (healthy and infected). Then the growth equation is

2.5 g(t)=r(1−A(t)),

where *r* is the host growth rate (constant, see electronic supplementary material, appendix 1 table S1).

The (time-dependent) rate at which leaf senescence occurs, is

2.6 $$\Gamma (t)=\left\{ \begin{array}{cc} 0.005\left(\frac{t-T_{GS61}}{T_{GS87}-T_{GS61}}\right)+0.1e^{-0.02(T_{GS87}-t)}, & \mathrm{if}\;t\geq T_{GS61},\\ 0, & \mathrm{if}\;t<T_{GS61}, \end{array}\right.$$

as in [12,14,18,30] (table 2). NB the rate of senescence of healthy host tissue does not depend on the amount of infected tissue.

#### 2.1.3. Mutation

We incorporate pathogen mutation, meaning that a small proportion of each pathogen strain’s offspring takes a different trait value. [30] used a Gaussian mutation kernel, assuming mutation events occur with probability *p*_*M*_ and with mutation scale *σ*^2^. Let *δ*(*x*) be the Kronecker delta (which is 1 when *x* = 0 and 0 otherwise). Then for a strain with trait value *j*, we denote the probability *P*(*k*, *j*) of its offspring taking trait value *k*

2.7 $$P(k,j)=(1-p_{M})\delta (k-j)+p_{M}\frac{1}{\sqrt{2\pi \sigma^{2}}}\exp(\frac{-{\left(k-j\right)}^{2}}{2\sigma^{2}}).$$

Any offspring predicted to take negative trait values are instead given trait value 0 and any that are predicted to take values greater than 1 are given trait value 1. This equation is illustrated in electronic supplementary material, appendix 1 figure S1.

Offspring with trait value *k* has a *per capita* transmission rate that is found by integrating over all possible parents with trait value *j*

2.8 $$\int_{0}^{1}\beta (j,t)I(j,t)P(k,j)\;dj.$$

For each parent with trait value *j*, we multiply *I*(*j*, *t*) by its infection rate *β*(*j*, *t*) and the probability of mutation from *j* to *k*; *P*(*k*, *j*). We integrate over all parents *j* ∈ [0, 1]. In the equation for d*S*(*t*)/d*t*, (equation 2.1), we integrate again to capture the *per capita* transmission of all strains in the population.

#### 2.1.4. Initial distribution of trait values

We use a gamma distribution for the curvature values (equivalent to −log(*k*)), to describe the initial distribution of trait values in the pathogen population (as in [30]). The gamma distribution is a two-parameter distribution—the default values are shown in electronic supplementary material, appendix 1 table S1. After each growing season, the population is normalized so that the total inoculum when integrating over all strains is *I*_0_ in the next season (electronic supplementary material, appendix 1 table S1). However, the relative amounts of each strain changes due to the fungicide applications.

#### 2.1.5. Yield

The model describes how the amount of healthy tissue and infected tissue changes throughout the season. To relate these quantities to the resulting crop yield, [30] fitted a generalized additive model to data relating disease severity (i.e. percentage of leaf area infected) to yield. Both quantities were measured at Growth Stage 75 [37], which is the model end time (table 2). Details of the model fitting process and implementation can be found in [30].

### 2.2. Qualitative resistance

The quantitative/polygenic resistance model we present can entirely capture the behaviour of a qualitative/monogenic system simply by choosing an initial pathogen distribution which has density at two *k* values only and setting the mutation proportion *p*_*M*_ to be 0. Note that this means that only selection is considered in this version of the model, since we only consider two strains and we would need another model fitting procedure to fit the appropriate mutation scale for a two-strain model. Reassuringly, the results of the model were found to be essentially identical when the mutation parametrization was changed in [30].

Denote these two pathogen strains *k*_*r*_, *k*_*s*_, at proportions *p*_*s*_, 1 − *p*_*s*_. Then the mean trait value is

2.9 $$\bar{k}=p_{s}k_{s}+(1-p_{s})k_{r}.$$

Using the model in this way allows us to directly compare the optimal dose choice for a qualitative system to a quantitative system, using the same model. We have complete flexibility over the choices of *k*_*r*_ and *k*_*s*_, which allows us to explore a range of different levels of partial resistance. Initially, we will explore the behaviour of the qualitative/monogenic system with partial resistance before comparing to the results of the quantitative system.

### 2.3. Quantitative resistance; parameter scan

In order to analyse when low doses perform better than high doses, we ran a parameter scan across 10 000 model runs. For this parameter scan, we used the full, quantitative version of the model (i.e. did not restrict the model to two strains alone). Further, the mutation proportion was not 0 for this scan, in contrast to the qualitative resistance results. Values were sampled randomly and independently for a variety of parameters, allowing us to explore the behaviour of the model for a range of parametrizations. For each run in the ensemble, we sampled values for: the two fungicide initial distribution parameters; the fungicide dose–response parameters (decay rate and asymptote); and the two mutation parameters (scale and proportion)—see table 3. We also calculate the initial mean fungicide effect at full dose, *ν*, which is calculated based on the pathogen distribution from the start of the first season (and averaged across all trait values *k*). This quantity depends on the fungicide distribution mean ($\bar{k}$) and asymptote (*ω*) as follows:

**Table 3.** Parameters/features involved in the parameter scan. The first six are randomly and independently sampled for each parameter run, while the mean fungicide effect at full dose (*ν*) is calculated based on these, and year varies between 1 and 35 in each run. The first four parameters are sampled from a uniform distribution, and the two mutation multipliers are sampled from a log-uniform distribution. The multipliers for decay rate, mutation scale and mutation proportion relate to the default values for Λ, *σ*^2^ and *p*_*M*_ (electronic supplementary material, appendix 1). In each case, the values of the parameters are scaled by the multiplier. For example, a sampled decay rate would be $\Lambda^{{\;}^{\prime }}=M_{d}\Lambda$.

symbol	description	range	equation
*μ*	pathogen gamma initial distribution mean (curvature)	[0, 25]	electronic supplementary material, appendix 3 equation 1
*b*	pathogen gamma initial distribution rate (curvature)	[0, 5]	electronic supplementary material, appendix 3 equation 1
*ω*	fungicide asymptote	[0, 1]	2.3
*M*_*d*_	fungicide decay rate multiplier	$\left[\frac{1}{3},3\right]$	(2.4)
*M*_*s*_	mutation scale multiplier	[0.1, 10]	(2.7)
*M*_*p*_	mutation proportion multiplier	[0.1, 10]	(2.7)
*ν*	mean fungicide effect at full dose	depends on *ω*, *μ*, *b*	(2.10), (2.11)
*Y*	year in model run	[1, 35] (but not sampled)	—

2.10 $$\nu =\omega \bar{k}+1-\omega .$$

**Figure 1.**
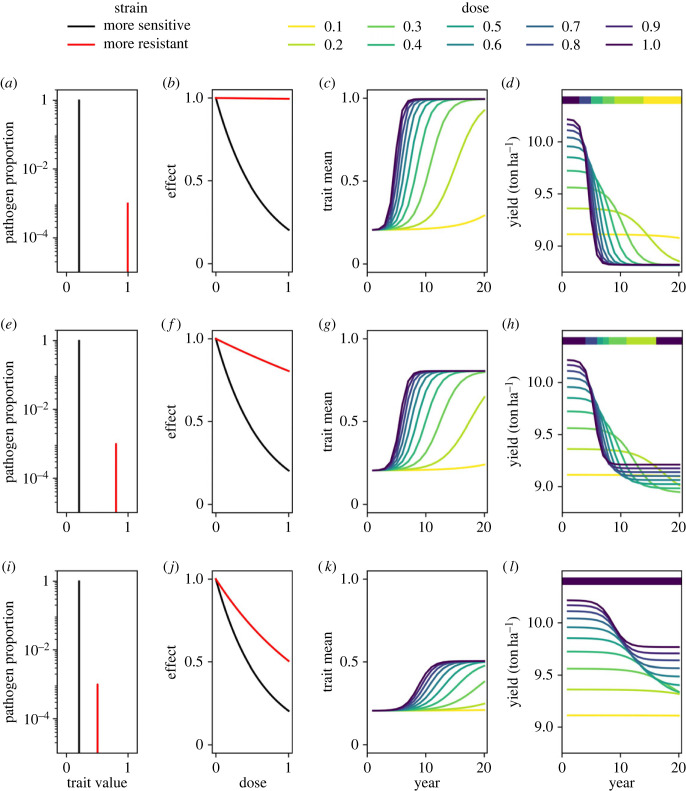
High doses can outperform low doses even in a qualitative resistance model. Each row corresponds to a single monogenic pathogen population evolving over 20 years. The left column shows the initial pathogen populations, each containing a sensitive and a resistant strain (*a*,*e*,*i*). Recall that *k* = 0 corresponds to a fully sensitive strain and *k* = 1 corresponds to a fully resistant strain. The middle-left column shows their corresponding dose–response curves (*b*,*e*,*j*). The middle-right column shows how the mean trait value (equation (2.9)) in the population changes as resistance develops for various different fungicide doses applied each year (*c*,*g*,*k*). The right column shows how the yield varies as resistance develops for the same dose choices (*d*,*h*,*l*). The colourbars at the top of panels *d*,*h*,*l* show which dose is optimal in each year. The top row (*a*–*d*) shows an example where lower doses are best for control/yield after the first few years. In the middle row (*e*–*h*), the optimal dose in any given year depends on how long it has been since the strategy began—in the very short and very long-term high doses are best but low doses are best in the intermediate period. In the bottom row (*i*–*l*), higher doses are best in all years. Parameter values. Resistant strain *k* value: *a*: 0.995; *e*: 0.805; *i*: 0.505. Sensitive stain *k* value: *a*,*e*,*i*: 0.205. Resistant strain initial density: 10^−3^. Mutation proportion: 0. Fungicide asymptote: 1.

Note that ‘effect’ here denotes the multiplicative effect of the fungicide on the pathogen’s infection rate. For example, *ν* = 1 means the pathogen is unaffected, whereas a low value of *ν* means that the pathogen is strongly suppressed by the fungicide. From equation (2.10), we see that if *ω* = 0, *ν* = 1 (i.e. the initial mean effect of the fungicide at full dose is 1, so that all strains are unaffected by the fungicide). However, if *ω* = 1, $\nu =\bar{k}$ (i.e. the initial mean effect of the fungicide at full dose equals the mean trait value—see electronic supplementary material, appendix 1 figure 1).

Note that $\bar{k}$ can be written in terms of *μ*, *b* (see electronic supplementary material, appendix 3)

2.11 $$\bar{k}=\frac{b^{\mu b}}{{\left(b+1\right)}^{\mu b}}.$$

For each run in the ensemble, we test out 10 doses from 0.1 to 1, allowing each dose strategy to be used for 35 years. The model returns the yield in each year, and in a given year *N* we are interested in the ‘best dose’, i.e. the dose which gives the highest yield in year *N* given the use of that dose in years 1, 2, …, *N*. The result of the scan was a dataframe containing one row per year of each ensemble member, along with the various variables described above and in table 3, and the best dose.

### 2.4. Gradient-boosted trees model

We fitted a gradient-boosted trees model in order to analyse the results of the parameter scan and seek relationships between the different features (i.e. the parameters in table 3) and the best dose. Gradient-boosted trees models are a machine learning technique based on an ensemble of decision trees. Each decision tree splits the input space into various disjoint regions, and the decision tree output is constant in any of these regions. The ensemble of decision trees is built up so that each successive model is fitted to the residuals of the previous ensemble model. When the loss function is the squared error, the residuals are proportional the derivative (gradient) of the loss function, hence the name gradient-boosting. The ‘learning rate’ (LR) is used to limit the impact of any individual tree within the ensemble by scaling the output by a value LR ∈ [0, 1]. This leads to much improved accuracy and better generalization to unseen data. We used the freely available python package XGBoost [34] to fit the gradient boosted regression model to the data. Gradient-boosted trees models give outstanding results across a wide range of applications; 17 of the 29 of the challenge winning solutions to Kaggle problems in 2015 used XGBoost [34]. They can be more accurate than neural networks and better at dealing with nonlinear, co-linear or strongly interacting variables than linear models [35].

#### 2.4.1. Model fitting

To fit the model, we split our data into two parts: a training and test set. These were split in an 80:20 proportion so that the first 8000 runs were used for model training. We optimized for the following hyperparameters (electronic supplementary material, appendix 2 tables S1, S2): max depth, number of estimators, learning rate, subsample, column sample by tree. This optimization was performed on the training set, using a fivefold cross-validation to assess the performance of each set of hyperparameters. We used the test set to check that the performance of the best model did not degrade significantly on completely unseen data. Choosing appropriate hyperparameter values helps avoid overfitting the model but ensures we retain sufficient complexity to capture the relationships found within the data. Model performance (in terms of root mean squared residuals) is shown in electronic supplementary material, appendix 2 table S3 and on predictions on (previously unseen) test data in electronic supplementary material, appendix 2 figures S1 and S2.

### 2.5. Shapley values

Shapley values [35,36] allow us to explain the output of our gradient-boosted trees model. They give an indication of how important each input feature is in controlling the value of a given output. Shapley values are a method from cooperative game theory which allow us to allocate credit to individual ‘players’ (i.e. model features in our case) for the output of the model (i.e. best dose in our case). The ‘game’ is the task of predicting a single instance (i.e. a single row) of the dataset given feature values. The ‘gain’ is the actual prediction for this instance minus the average prediction for all instances. A ‘coalition’ is a subset of players/features. The Shapley value of a particular feature is a way of measuring that feature’s contribution to the model output, i.e. that player’s contribution to the outcome of the game. For a given feature value and coalition excluding that feature, we can find the marginal contribution of that feature to the prediction when it is added to the coalition, by comparing the prediction from the coalition size *k* averaged over all possible values of the *N* − *k* excluded variables to the prediction from the coalition size *k* + 1 that includes the feature. Then the Shapley value is the (weighted) average over all marginal contributions of that feature value, i.e. the average (over all coalitions excluding the feature) of the change in prediction that occurs when that feature value is included in the coalition. They are a valuable tool facilitating interpretability and explainability of complex and otherwise opaque models.

In general, Shapley values can only be approximated since computing them exactly gets very computationally complex/expensive as the number of variables included increases. However, for gradient-boosted trees, Shapley values can be calculated exactly, and in polynomial time [35]. We use the open-source Python package ‘shap’ to do so. This helps us analyse the gradient-boosted trees model output and further understand the relationship between the various fungicide parameters and the corresponding best dose across the full range of time scales considered (1–35 years).

## 3. Results

### 3.1. Can high doses ever be best with qualitative partial resistance?

If resistance is complete (i.e. the *k* value of the resistant strain is *k*_*r*_ = 1), lower doses are preferable in terms of yield/control to high doses (figure 1*a*–*d*), apart from in the very early years when the resistant proportion is at a very low density. This is because higher doses select more strongly for the resistant strain (figure 1*c*), and the fungicide is completely unable to control the resistant strain, leading to reduced yields from higher doses once resistance has developed (figure 1*d*). However, if resistance is partial, there are cases where high doses are better over certain time frames (figure 1*e*–*h*). In the very short and very long term, the pathogen population is almost entirely sensitive and (partially) resistant respectively. In both these cases, higher doses offer better control. The trade-off occurs in the intermediate period where resistance develops more quickly (figure 1*g*) to the higher dose fungicide programmes, leading to lower yield for higher doses in this intermediate period of time (figure 1*h*). When the partial resistance is weak (figure 1*i*), it is possible for high doses to always outperform low doses (figure 1*i*–*l*). Although higher doses still give more rapid selection in this scenario (figure 1*k*), the increase in resistance does not outweigh the increased control offered by higher doses (figure 1*l*).

**Figure 2.**
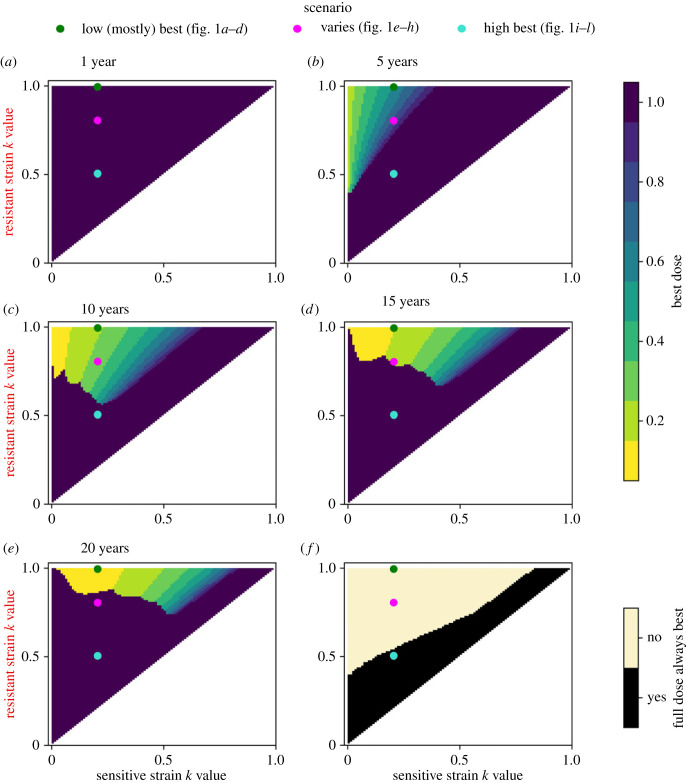
When do high doses outperform low doses in the qualitative resistance model? The resistant strain was constrained to take a higher *k* value than the sensitive strain, leading to the upper left triangle in each heatmap (recall that *k* = 0 is fully sensitive and *k* = 1 is fully resistant). We denote the three scenarios from figure 1 by the three dots: green relates to figure 1*a*–*d*; pink relates to figure 1*e*–*h*; turquoise relates to figure 1*i*–*l*. Yellow/green regions denote where using the low dose every year leads to a better yield in that year than using full dose every year, and (dark) purple where full dose is better. For some pairs of strains, high doses always give the best yield (*f*). Parameter values. Resistant strain initial density: 10^−3^. Mutation proportion: 0. Fungicide asymptote: 1.

To systematically explore when partial resistance affects optimality of high doses, we run a scan over the possible *k* values that the sensitive and resistant strains could take (figure 2). For each year *Y*, we plot the best dose, i.e. the one which gives the highest yield in year *Y* given the use of that dose in years 1, 2, …, *Y*. The doses considered are 0.1, 0.2, …, 1. In figure 2, we consider *Y* = 1, 5, 10, 15, 20.

In year 1, high doses are best for all strain pairings since they offer better control and the resistant frequency is low (figure 2*a*). As time progresses, the best dose depends on the *k* values of the resistant and sensitive strains (figure 2*b*–*e*).

The three scenarios from figure 1 are shown in figure 2 by the green, pink and turquoise dots. The green dot shows when the resistant strain is fully resistant (i.e. figure 1*a*–*d*), but the pink (figure 1*e*–*h*) and turquoise (figure 1*i*–*l*) show when the resistance is partial (weaker in the turquoise dot example). In the green dot example (*k*_*s*_ = 0.205, *k*_*r*_ = 0.995), low doses give higher yields for all years from year 5 onwards (figure 2*b*–*e*), although note that full dose is better in year 1 (figure 2*a*). In the pink dot example (*k*_*s*_ = 0.205, *k*_*r*_ = 0.805), full dose gives higher yields initially and after many years (figure 2*a*,*e*), but the lower dose is better in the interim period (figure 2*b*–*d*). This is because full dose is better when the population is (almost) completely sensitive or completely resistant, but in the interim period the resistant proportion increases more rapidly when full dose is used. If the resistant strain is only weakly resistant (turquoise dot; *k*_*s*_ = 0.205, *k*_*r*_ = 0.505), then full dose always performs better or equal to the lower dose (figure 2*a*–*f*).

When the resistant strain is very close in *k* value to the sensitive strain, high doses are always better since in this case the increased selection for resistance has a smaller effect than the increased control offered by a high dose (figure 2*f*; pairs of values of *k* which are nearly equal are just above the line *k*_*s*_ = *k*_*r*_, which is shaded in black). When resistance is complete (i.e. *k*_*r*_ = 1), and the sensitive strain is highly sensitive (*k*_*s*_ ≈ 0), the low dose is better by year 5 (figure 2*b*). In later years (figure 2*c*,*d*,*e*), the population is essentially completely resistant, so full dose and the low dose both perform badly, offering very little control.

When the resistant strain is partially resistant (*k*_*r*_ < 1), then once enough time has passed that (virtually) the entire population is resistant, the full dose strategy outperforms the low dose strategy (since higher doses can control the partially resistant strain better). This means that after sufficiently many years, high doses will be best if resistance is partial (*k*_*r*_ < 1). However, the time taken to reach this entirely resistant stage is shorter with high doses due to increased selection from the full dose strategy, and the yield obtained when the population reaches this point may be unacceptably low. Depending on the two *k* values, there may be an interim period where low doses are better than high (yellow/green in figure 2).

### 3.2. When are high doses best in the quantitative resistance model?

We now explore which doses give the best yield over time in the quantitative resistance model. Understanding the behaviour of the qualitative resistance model is useful since it explains the behaviour of pairs of strains taking different *k* values (figures 1 and 2). The quantitative resistance model is more complex, with many different strains all taking different *k* values and all competing with each other.

**Figure 3.**
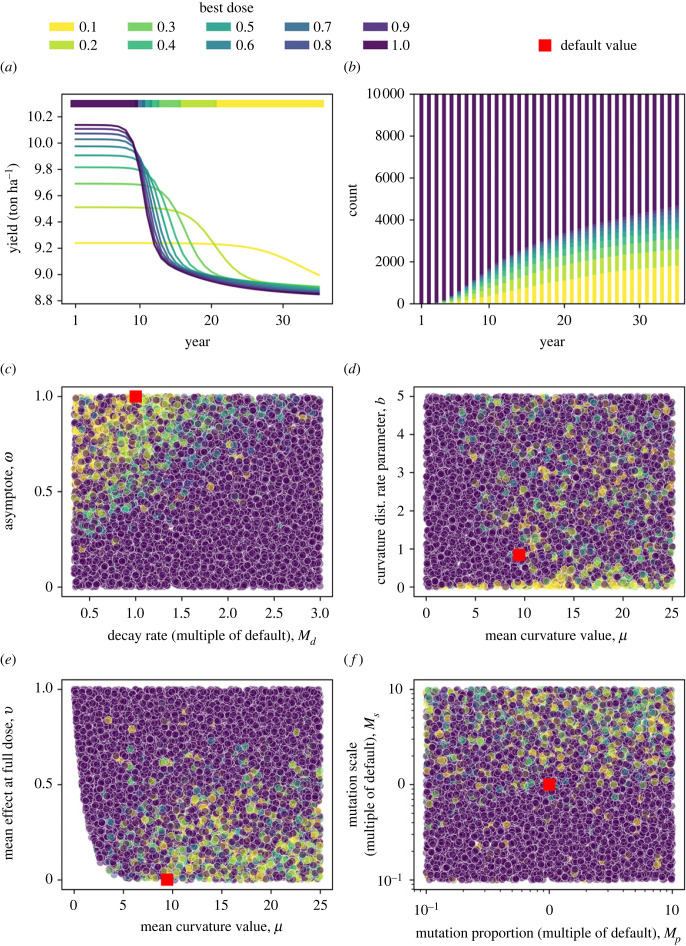
High doses can outperform low doses in the quantitative resistance model. Panel (*a*) shows one example model run from the ensemble. The ‘best dose’ in any given year is the dose which gives the highest yield in that year, as shown by the colourbar at the top of (*a*). Panel (*b*) shows how frequently higher doses outperformed low doses depending on the time scale of interest. Initially, high dose is invariably best since the population is still highly sensitive, but after some time low doses are better for some of the parameter combinations generated in the scan. Panels (*c*–*f*) explore which parameters led to low doses outperforming high doses, focusing on year 10 only (arbitrarily chosen). This helps us understand which parameters drive the effect. Higher efficacy fungicides, i.e. with a high dose–response asymptote (*c*), a low decay rate (*c*), a high mean curvature value (*e*) or a low mean fungicide effect at full dose (*e*) require lower doses. Note that the mean fungicide effect at full dose *ν* is a function of *μ*, *b* and *ω* as described in equations (2.10) and (2.11), which results in the shape of the space in panel (*e*). Populations with greater mutation scales require lower doses (*f*), but the mutation proportion is less important. There is less of a clear pattern in (*d*), although low doses seem better for high values of mean curvature *μ* and for low values of the rate parameter *b*. Default values as in [30] are denoted by red squares. Parameter values for panel (*a*) are in electronic supplementary material, appendix 1 table S2, and a plot of how the pathogen distributions from (*a*) change with different doses is in electronic supplementary material, appendix 3 figure S3.

We present the results of the parameter scan, which involved an ensemble of 10 000 model runs. For any given model run (e.g. figure 3*a*), we can find the best dose in each year by comparing yield in that year for each of the 10 different doses (0.1,0.2,…1) tested.

In the example shown, initially high doses are best but in later years lower doses are favoured (figure 3*a*). This behaviour occurred in some model runs, but for many full dose was always favoured (figure 3*b*). After 10 years, lower doses tend to be best for lower decay rates and higher fungicide asymptote parameters, corresponding to higher efficacy fungicides which remain at high concentrations for longer (figure 3*c*). For a given mean curvature value, a higher ‘rate’ parameter (*b*) means decreased variance in the distribution of curvature values. Note that the rate parameter *b* influences the shape of the initial pathogen trait distribution and despite its name it is not an infection rate or a parameter with any time-dependence. Higher mean curvature values and lower rate parameters tend to incentivize lower doses (figure 3*d*). The effect of the gamma distribution parameters on the resulting trait distribution is shown in electronic supplementary material, appendix 3 figures S1 and S2. The lower the mean fungicide effect at full dose, corresponding to higher efficacy fungicides which suppress the disease more strongly, the more likely that lower doses will be best (figure 3*e*). Higher mutation scales tend to correlate with lower doses being preferable, but the mutation proportion is less important (figure 3*f*).

**Figure 4.**
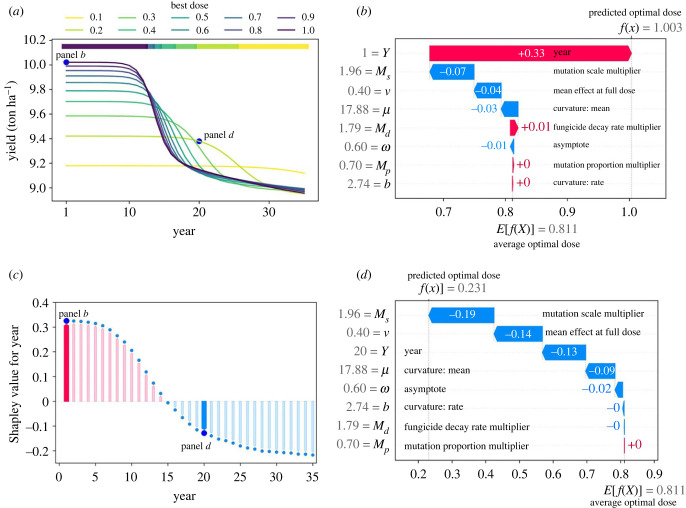
Introducing Shapley values. Shapley values give an indication of how important each input variable is in controlling the optimal dose (i.e. the output of the gradient-boosted trees model). The optimal dose in any given year varies in this single model run from the ensemble of 10 000 (*a*). The first year and 20th years are highlighted since these are the data points shown in more detail in (*b*,*d*), respectively. Initially, full dose is best, but in later years lower doses are optimal. Panel (*b*) is a ‘waterfall plot’ which explains a single model prediction corresponding to the 1st year from this model run. In this example, the year (*Y*) is the most important feature and the predicted best dose is 1.003 (i.e. full dose with a small model error). See table 3 for an explanation of the features and their ranges. The grey numbers indicate the values of each feature (e.g. the year *Y* = 1 and the mutation scale multiplier *M*_*s*_ = 1.96), and the arrows indicate that feature’s effect on the model output (i.e. whether the value taken acts to increase or decrease the predicted optimal dose). Panel (*c*) shows, for this model run, how the year feature influences the model output. Higher doses are better in early years, which is reflected in positive contributions when the year is small and negative contributions when the year is larger than 15. Again we highlight the first and 20th years since these relate to (*b*) and (*d*), respectively. (*d*) A waterfall plot for the 20th year from this model run. The predicted best dose is 0.231 and the most important feature is the mutation scale (*M*_*s*_) which decreases the predicted best dose by 0.19. Parameter values for (*a*) are in electronic supplementary material, appendix 1 table S2. See the main text for an explanation of how Shapley values are calculated.

We use Shapley values to explain the influence of different model parameters/variables on the optimal dose, depending on the time scale. The optimal dose in year *N* is the one which, when applied every year, gives the best yield after *N* years. Shapley values sum to the difference between the baseline/expected model output and the current model output for a particular prediction being explained. Each variable in each year of each model run has a single Shapley value. For another single model run, we can see how the yield varies for different doses (figure 4*a*). The waterfall plots in figure 4*b*,*d* each show a single prediction from the model, corresponding to the first year of this model run and the 20th year, respectively. The gradient boosted model uses the explanatory features (table 3) to predict which dose is optimal. The Shapley values explain how the prediction differs from the expected optimal dose of 0.811 (averaged across the entire dataset). Features are added in one at a time until we reach the model output of 1.003 in the first year (i.e. best dose is full dose, plus a small model error). Note that the model output is a continuous variable, so in general it won’t equal a multiple of 0.1. The main contribution in year 1 is from the year variable *Y*—the best dose to use in the first year is full dose since it provides improved control in that year and resistance is minimal. For this model run, we plot the impact of the year variable figure 4*c*. Here, we plot the feature value (year, *x*-axis) against the corresponding Shapley value (*y*-axis). As time progresses the Shapley value in each year of this model run decreases, meaning that higher doses are best in early years but lower doses are best if longer time scales are considered. In the 20th year, mutation scale (*M*_*s*_) and mean fungicide effect at full dose (*ν*) are the variables with the largest contributions (figure 4*d*). The best dose predicted by the gradient-boosted trees model for the 20th year is 0.231. In the actual model run (*a*), 0.2 was the best dose in year 20 and 0.3 was the best dose in year 19.

**Figure 5.**
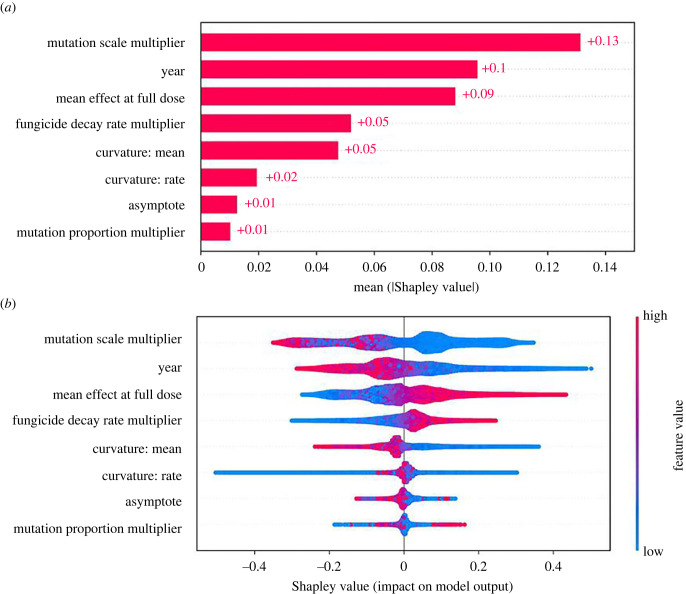
Which variables have the greatest impact on determining the optimal dose? Panel (*a*) shows feature importance, measured in terms of the mean absolute Shapley value over all given samples. Mutation scale *M*_*s*_, year *Y* and fungicide efficacy (mean fungicide effect at full dose *ν*) are the most important features. Panel (*b*) shows the effect of the most important variables on the model output across the scan. Each point corresponds to a single year from a single model run. Blue points for any given variable correspond to low values for that variable, and similarly pink points represent high values for that variable. Their impact on the model output is shown on the *x*-axis. The density of points is represented by their height (comparable to a violin plot). Panel (*b*) shows a more detailed explanation of the impact of each variable on the model output than (*a*).

Feature importance is shown in figure 5*a*, and summarizes the influence of each parameter on the optimal dose. This is measured in terms of the mean absolute Shapley value over the entire dataset. Figure 5*b* shows the relationship between the features and the Shapley value in more detail. Each year of every model run is represented by a single point for each feature. Higher values for each feature are shown in blue, and lower values in pink. The larger the absolute Shapley value, the bigger the impact of that point on the model output. The most important variables are the mutation scale (*M*_*s*_), the year (*Y*) and the mean fungicide effect at full dose (*ν*) (table 3).

**Figure 6.**
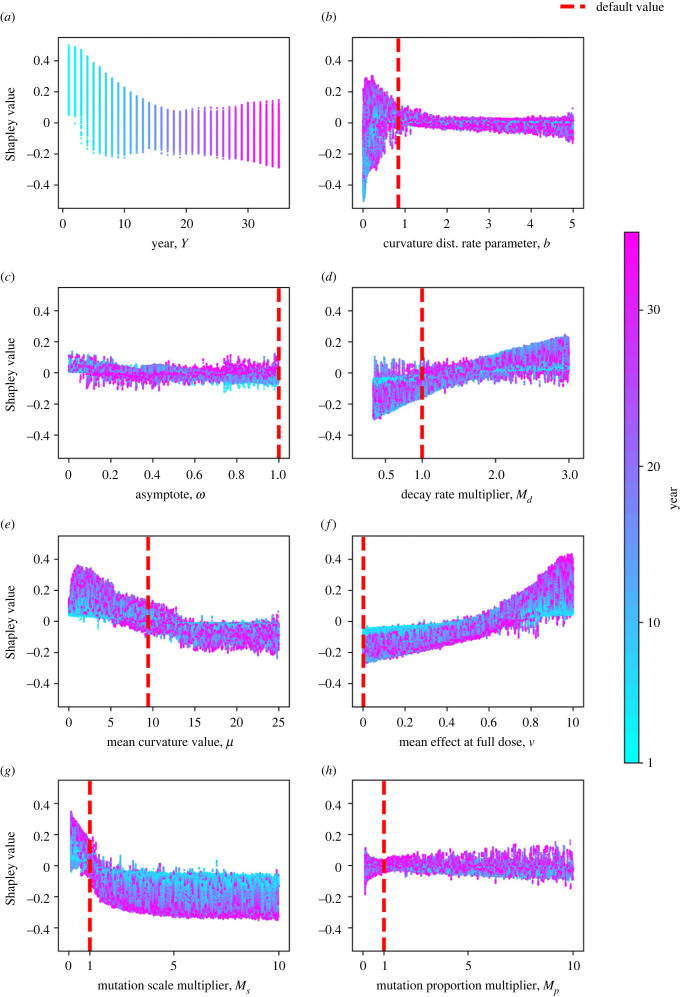
Which variables correlate with optimality of high doses? Here, we show the effect of many of the model variables on the output. Each point corresponds to a single year from a single model run in the ensemble. Each *x*-axis value corresponds to a variable value, and each *y*-axis is the Shapley value for that feature, corresponding to how much that feature contributes to the model output for that specific prediction. A high Shapley value corresponds to that variable changing the output to a higher dose being optimal. Default model values as fitted in [30] are shown by the red dotted lines. The colours help indicate interactions between each feature and the feature ‘year’ (*Y*). The model predicts higher doses for decreased year (*a*) and decreased fungicide efficacies, i.e. higher decay rates (*d*), lower mean curvatures (*e*), and lower values of the mean fungicide effect at full dose for the initial pathogen distribution (*f*). However, the model predicts higher doses for increased decay rates (*d*), mutation scales (*g*) and proportions (*h*). The curvature rate parameter *b* (*b*) and mutation proportion multiplier (*h*) features have a smaller effect, although very small values of *b* have a more significant effect. This is because they relate to higher pathogen density at very low and very high *k* values (see electronic supplementary material, appendix 3). The asymptote variable *ω* also has a relatively small effect (*c*), most likely because its impact on the model is via its impact on the mean fungicide effect at full dose *ν*.

To further understand the effect of each variable, we plot the Shapley value against each parameter value showing the results for all members of our ensemble (figure 6). This is the same style of plot as figure 4*c*, but we show all data from all model runs rather than only those from a single model run. In all panels, we use colour to show the interaction with the year (NB the colours in figure 6*a* do not relate to an interaction since the variable plotted in figure 6*a* is year). To further understand the effect of the model parameters without the year interaction, we fit a model to the data from year 10 only and performed similar analysis in electronic supplementary material, appendix 4, and found that the order of feature importance remains the same, apart from the mean fungicide effect at full dose and decay rate features swapping importance order from second and third most important to third and second most important.

The predicted best dose decreases strongly with the year and mutation scale (figure 6*a*,*g*) but increases strongly with the decay rate and the mean fungicide effect at full dose (figure 6*d*,*f*). This matches the result shown in figure 5*a* showing the most important four features as summarized by average absolute Shapley value to be mutation scale, year, mean fungicide effect at full dose and decay rate. Higher mean curvature values *μ*, (corresponding to higher efficacy fungicides) mostly correspond to lower doses being optimal (figure 6*e*). The asymptote feature, *ω*, has quite a small impact on the model (figure 6*c*), most likely since its impact is essentially summarized by the mean fungicide effect at full dose feature *ν*. The curvature rate parameter *b* has a small effect when it is greater than 1 but a large, though unpredictable effect when it is smaller than 1 (figure 6*b*). This is because the effect of increased variance in the pathogen distribution interacts strongly with other variables, and leads to large changes in the initial pathogen distribution with high density near *k* = 0 and *k* = 1 (electronic supplementary material, appendix 3 figure S1). The mutation proportion multiplier has a smaller effect than the mutation scale (figure 6*h*).

Many of the variables interact strongly with year. Notably the mean curvature value (*μ*, figure 6*e*), the mean fungicide effect at full dose (*ν*, figure 6*f*) and the mutation scale (*M*_*s*_, figure 6*g*) have an exaggerated effect on the model in later years but a much smaller effect in early years. This is because high doses are favoured in early years but there is bigger variation in possible outcomes in later years depending on whether low doses begin to outperform high doses.

#### 3.2.1. Biological interpretation

**Figure 7.**
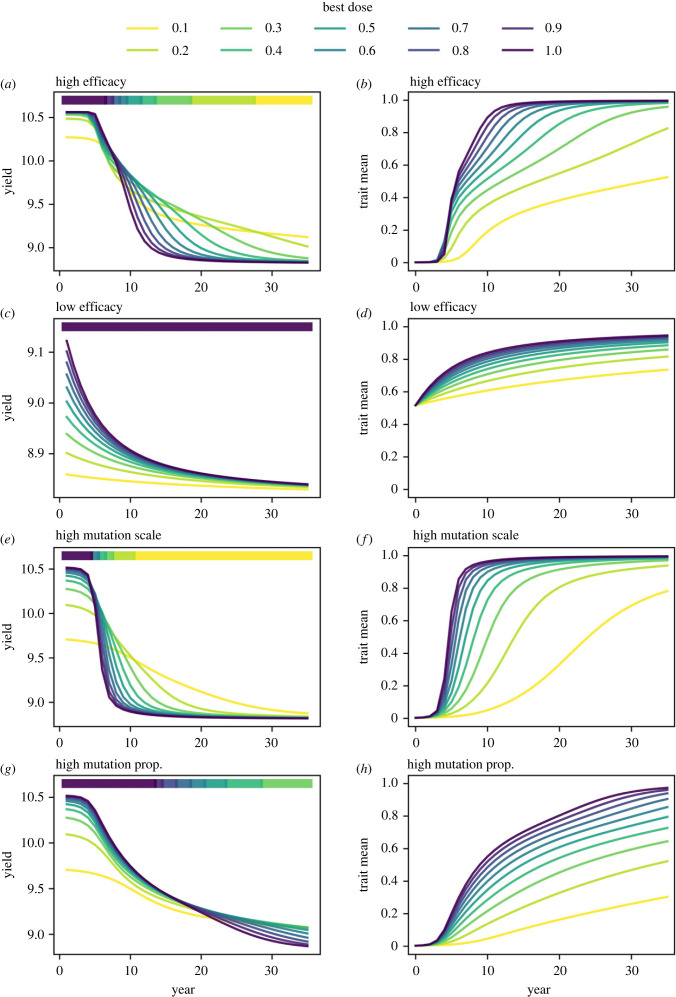
Why does fungicide efficacy and pathogen mutation affect the dose recommendation? Here, we present four scenarios along each of the four rows, as per the panel labels. High doses of high efficacy fungicides select strongly for resistance, leading to lower doses being optimal after only a few years (*a*,*b*). However, higher doses of lower efficacy fungicides can provide more effective control without selecting too rapidly for resistance (*c*,*d*). Large mutation scales lead to rapid resistance development when high doses are used, incentivizing lower doses (*e*,*f*). Large mutation proportions do not lead to as rapid resistance development if the mutation scale is not particularly large (*g*,*h*). In this scenario, it is possible for high doses to be best for many years. Parameter values: see electronic supplementary material, appendix 1 table S3.

The parameter scan suggests that higher efficacy fungicides tend to need lower doses for optimality, after the first few years in which high doses are always best (figures 3, 5 and 6). This is because the selection for resistant strains is stronger. If the initial mean fungicide effect at full dose (*ν*) is very low, corresponding to a higher efficacy fungicide, then the difference in infection rates between sensitive and resistant strains is larger due to stronger suppression of sensitive strains. This means resistance develops more quickly, in accordance with the so-called governing principles [25]. This means low doses are preferable to higher doses for medium or longer time scales (figure 7*a*,*b*). Making a comparison with the monogenic case (figure 2), lower efficacy fungicides have the sensitive strain(s) at a relatively high *k* value(s), away from *k* = 0, which tends to incentivize higher doses unless the resistant strain is close to *k* = 1. This means that in most cases lower efficacy fungicides correlate with higher doses being optimal (figure 7*c*,*d*). However, if the mutation scale is very high (i.e. mutations tend to lead to a larger change in offspring phenotype) then the pathogen population reaches higher density near *k* = 1 quicker (i.e. becomes highly resistant quicker, see figure 7*e*,*f*), unless doses are lowered greatly which reduces selection. Note that the increasing the mutation proportion (i.e. the proportion of mutant pathogen offspring) has a smaller effect than increasing the mutation scale (figure 7*g*,*h*).

In all examples shown, higher doses give increased selection for resistance (figure 7*b*,*d*,*f*,*h*). However, this does not guarantee reduced control and correspondingly reduced yield if the difference in selection is small relative to the increase in control offered by a higher dose (compare figure 7*a*,*e*,*g* with figure 7*c*).

## 4. Discussion

Fungicide dose choice can be important in determining how quickly resistance develops [24]. Although the existing literature suggests that higher doses contribute to increased selection [12,13,18,24,25], many studies on optimal dosage neglect partial resistance, and to the best of our knowledge no modelling study considers the case of quantitative resistance. The model used in this study addressed complete or partial qualitative resistance as well as quantitative resistance. Note that in the case of qualitative resistance we exclude mutation, but in the case of quantitative resistance we include mutation. We do not believe this to be important to the model results, since a range of outcomes (i.e. whether higher or lower doses produce optimal yields) are shown to be possible in both cases. We show that although higher doses can often lead to increased selection for resistance in these scenarios, the control offered by higher doses may still outperform that offered by lower doses, both when resistance is qualitative and partial (figures 1 and 2) and when resistance is quantitative (figures 3 and 4).

We tested the performance of different fungicide application programmes which use fixed doses (i.e. same dose repeatedly used each year). We sought the optimal fixed dose to maximize yield in the final year of a fungicide programme, depending on the length of the programme, and on other model parameters. In a very short fungicide programme (i.e. 2 or 3 years), high doses always gave the best control. However, over longer time scales the optimal dose choice depends on model parameters (figures 4–6). Note that this does not mean high doses should always be used in the early years of a fungicide programme. Rather, it means that if the time scale of interest is very short, high doses every year will give better yields than low doses every year.

High fungicide efficacies often incentivize lower doses. This can be in terms of the decay rate of the fungicide itself, or in its interaction with the pathogen via the dose response curve: the asymptote, mean curvature or (initial) mean fungicide effect at full dose can be implicated—see figure 6*c*–*f*. This is because the difference in infection rates between resistant and sensitive strains is greater with high efficacy fungicides, leading to greater selection (figure 7*a*–*d*) according to the governing principles as originally developed for monogenic resistance [25]. In figure 7*a*,*b,* the rapid selection for resistant strains outweighs the benefit in control offered by increased dose, whereas in figure 7*c*,*d* the difference between the resistant and sensitive strains is smaller, so selection is less exaggerated and the resistance does not outweigh the benefit to control offered by increasing fungicide dose.

Increased pathogen mutation scales also incentivize lower doses (figure 6*g*). This is because increased mutation scales can rapidly lead to higher densities of highly resistant strains. These strains are selected for more strongly if doses are higher, so low doses are best in this case (figure 7*e*,*f*). However, increased mutation proportions may not have the same effect unless mutation scales are also increased (figures 6*h* and 7*g*,*h*).

The results characterised the optimal dose solely in terms of instantaneous yield, i.e. the yield in any given year, rather than the total yield up until that point. Using instantaneous yield ignores the magnitude of the difference between the optimal yield and sub-optimal yields. This difference may be much larger in some years than others. To explore the effect of optimizing for the total yield rather than instantaneous yield, we ran a similar analysis combining a gradient-boosted trees model with Shapley values (electronic supplementary material, appendix 5). The results were broadly similar, although the initial (sometimes large) benefit to using high doses meant that there were more parameter combinations which favoured high doses (electronic supplementary material, appendix 5 figures S1 and S2). The most important explanatory variables were similar, although year became less important than the effect of the decay rate multiplier *M*_*d*_ (electronic supplementary material, appendix 5 figures S3 and S4).

In [17], dose–response convergence [24] is used to explain the potential for higher doses to delay emergence of resistance when resistance is monogenic and partial (and affects the ‘slope’ of the dose–response rather than the asymptote, i.e. ‘Type 2’ partial resistance). Dose–response convergence means the difference in infection rates between sensitive and resistant strains is smaller at high doses than at lower doses (see electronic supplementary material, appendix 1 figure S1B). For some models, this can theoretically lead to higher doses exerting lower selection pressure on resistant strains. In this work, resistance is characterised in terms of the curvature parameter, making resistance in the model analogous to Type 2 partial resistance. However, we use a different dose–response curve parametrization and assume that the fungicide concentration decays exponentially (following [12,13,18,38]) rather than remaining constant. The decay in fungicide concentration largely negates the dose–response convergence argument (ignoring density-dependent effects). This is because even if selection is lower when a high dose is applied, there will be stronger selection as the chemical decays because the concentration passes through all lower doses. This means that the selection for resistant strains is greater for higher doses, even in the quantitative resistance case. However, dose–response convergence may limit the extent to which selection increases as dose increases. This is illustrated by the model in electronic supplementary material, appendix 6, which shows that higher doses dominate less often when resistance is characterised by the fungicide asymptote parameter (analogous to partial resistance Type 1). Unlike [17], our focus was on the yield obtained from using different doses, rather than the effect of dose choice on emergence of resistance. This means we take into account the benefit in control from using a higher dose. In the quantitative resistance case, the optimal yield was from full dose for over 5000 model runs (from the ensemble of 10 000 in the randomization scan) in every single model year (figure 3*b*).

Lundberg [13] show that, for mixtures of fungicides to which resistance is qualitative and partial, substantially higher than minimal doses maximized lifetime yield (time for which acceptably high yields can be maintained), particularly if the partial resistance was Type 2 partial resistance. Conversely, van den Bosch *et al.* [24] state that dose–response convergence is unlikely to be relevant to single-site fungicides (i.e. the monogenic/qualitative resistance case), since dose–response convergence is unlikely to occur within a legal range of doses when there is a single genetic change which confers a high level of resistance. However, they suggest it may be relevant for azole fungicides (i.e. quantitative resistance). Our results suggest that high doses still tend to lead to greater levels of resistance in the quantitative resistance case, again due to exponential fungicide decay negating the dose–response convergence argument. Nevertheless, for both quantitative and partial qualitative resistance, this increased selection may not be sufficient to make low doses optimal in terms of yield, since the control offered by increasing dose may outweigh the increased selection if resistance is not complete.

Various modelling assumptions were made when deriving the epidemiological model. Fitness costs are neglected, which may have a significant impact [39]. However, [17] did not find fitness cost to have any major effect on their results on emergence time for fungicide resistance. We also neglect to model Septoria’s latent period [40] for simplicity. Although we consider emergence of new strains in the population via mutation, there is no demographic stochasticity, meaning that strains at low densities have zero probability of dying out. Further, the mutation kernel is Gaussian, although larger jumps in trait value may be possible. However, we had no empirical data to inform an alternative parametrization. Further, the mutation scale was shown to have limited effect on the results of this model [30]. Demographic stochasticity could be important to include since one of the existing arguments for higher doses is delaying emergence of resistance via reducing the population size and therefore reducing the absolute number of mutant offspring [24]. For simplicity, spatial effects are ignored, although future work may consider them given the potential for fungicide strategies based on spatial risk [41].

Although we consider both Type 1 and Type 2 partial resistance, we do not allow both to occur simultaneously, i.e. resistance is characterised in terms of the curvature parameter (main text) or the asymptote parameter (electronic supplementary material, appendix 6) but never in terms of both. Future work could consider a model where resistance is characterised in terms of both dose–response parameters. However, this would require a much more complex model fitting procedure with significantly more data, because the initial proportion of the pathogen population with every pair of possible curvature and asymptote values would need to be characterised.

Previously Sobol’s indices were used to provide a global sensitivity analysis of a plant disease model [33]. We presented an alternative method, relying on a gradient-boosted trees model and Shapley values. The gradient-boosted trees model performance on the test set was 0.106 (root mean squared error, see electronic supplementary material, appendix 2). This approach allows us to isolate the effect of the different variables (figures 4–6). This method allows for local explanations of the impact of parameters in different regions of parameter space, rather than just a global measure of feature importance. We then tested the biological insights from this analysis in the original epidemiological model (figure 7). Future work could explore the uncertainty of each Shapley value, potentially by using an ensemble of models to give uncertainty for each prediction. However, we have shown that the gradient-boosted trees/Shapley values method can explain the output of an epidemiological model, which can help identify useful biological relationships.

Although the model in [30] is capable of addressing disease-resistant cultivar control, for simplicity we omitted it here. However, it would be interesting to explore whether introducing cultivar control had any effect on the optimal dose. In early years lower doses could be used while retaining acceptable control if disease-resistant cultivars were used, but equally higher doses could offer increased protection to the host, delaying the breakdown of cultivar control [42]. Much of the fungicide resistance modelling literature is concerned with mixtures containing fungicides challenged by qualitative resistance [12,13,18]. The model could be extended to help finding optimal doses depending on initial conditions and the dose–responses of the fungicides in the mixture in the quantitative resistance case, or the case where one fungicide faced qualitative resistance and the mixing partner faced quantitative resistance.

It would be interesting to explore time-variable tactics. For example, in earlier years acceptable control could be obtained using low doses, and dose only increased once necessary to achieved desired levels of control. This would provide a strategy which achieves excellent control while vastly reducing selection for resistance. This approach is particularly useful in the case of quantitative resistance, since higher doses can still provide some control when resistance arises, unlike the case of complete qualitative resistance. Implementing a time-variable strategy would require an understanding of the state of the pathogen population each year. This is difficult to accurately estimate, because the effect of the fungicide on different pathogen strains in this model is parametrized in terms of the impact on growth rate, whereas data on pathogen sensitivity/resistance is usually measured in terms of the concentration of fungicide required to reduce the population by 50%. However, it is relatively simple to estimate the population mean based on the average control achieved (similar to the model fitting process in [30]). Using control data over several consecutive years could provide an estimate of the distribution of pathogen trait values/curvatures (by approximating the pathogen distribution with a smooth parametrized probability distribution, for example the gamma distribution as in [30]).

An economic analysis accounting for fungicide costs would also be interesting [43,44]. This approach would help us understand how to delay resistance development while maintaining good crop and economic yields for as long as possible. Nevertheless, by showing how both high and low doses can lead to optimal yields over different time scales, as well as understanding the drivers of this behaviour, we have shown how optimal dose choices for quantitative and/or partial qualitative resistance may frequently differ from optimal choices when resistance is qualitative and complete.

## Data Availability

An implementation of the model in the freely available programming language Python (Python Software Foundation, available at python.org) is online at github.com/nt409/quant-res-doses. The data are provided in electronic supplementary material [45].
